# Immortalized human myoblast cell lines for the delivery of therapeutic proteins using encapsulated cell technology

**DOI:** 10.1016/j.omtm.2022.07.017

**Published:** 2022-08-01

**Authors:** Aurelien Lathuiliere, Remi Vernet, Emily Charrier, Muriel Urwyler, Olivier Von Rohr, Marie-Claude Belkouch, Valentin Saingier, Thomas Bouvarel, Davy Guillarme, Adrien Engel, Patrick Salmon, Thomas Laumonier, Julien Grogg, Nicolas Mach

**Affiliations:** 1 Oncology Division, Geneva University Hospital and Medical School, 1211 Geneva, Switzerland; 2 Centre for Translational Research in Onco-Hematology, Oncology Division, Geneva University Hospital and University of Geneva, 1211 Geneva, Switzerland; 3 MaxiVAX SA, 1202 Geneva, Switzerland; 4 Institute of Pharmaceutical Sciences of Western Switzerland (ISPSO), University of Geneva, 1211 Geneva, Switzerland; 5 School of Pharmaceutical Sciences, University of Geneva, 1211 Geneva, Switzerland; 6 Department of Basic Neurosciences, University of Geneva, 1211 Geneva, Switzerland; 7 Cell Therapy and Musculoskeletal Disorders Laboratory, Department of Orthopaedic Surgery, Faculty of Medicine, Geneva University Hospital and University of Geneva, 1211 Geneva, Switzerland

**Keywords:** encapsulated cell technology, cell engineering, macroencapsulation, drug delivery, allogeneic cell transplant, myoblast, cell therapy, ex vivo gene therapy

## Abstract

Despite many promising results obtained in previous preclinical studies, the clinical development of encapsulated cell technology (ECT) for the delivery of therapeutic proteins from macrocapsules is still limited, mainly due to the lack of an allogeneic cell line compatible with therapeutic application in humans. In our work, we generated an immortalized human myoblast cell line specifically tailored for macroencapsulation. In the present report, we characterized the immortalized myoblasts and described the engineering process required for the delivery of functional therapeutic proteins including a cytokine, monoclonal antibodies and a viral antigen. We observed that, when encapsulated, the novel myoblast cell line can be efficiently frozen, stored, and thawed, which limits the challenge imposed by the manufacture and supply of encapsulated cell-based therapeutic products. Our results suggest that this versatile allogeneic cell line represents the next step toward a broader development and therapeutic use of ECT.

## Graphical abstract

**Figure:**
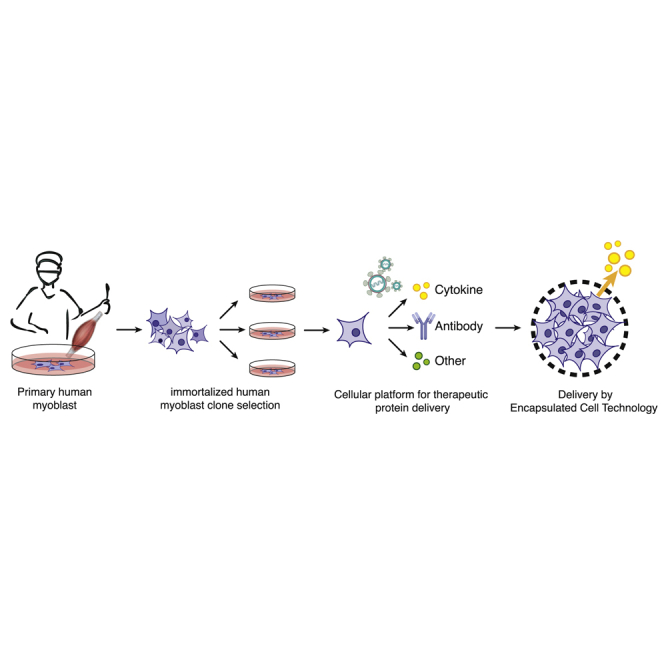

## Teaser

In this manuscript, Lathuiliere and colleagues describe the development and characterization of human myoblast cell lines tailored for the stable delivery of therapeutic proteins using encapsulated cell technology (ECT). These cell lines could potentially accelerate the clinical development of ECT.

## Introduction

Encapsulated cell technology (ECT) is an effective approach for the continuous and local delivery of therapeutic proteins released by genetically modified cells. In the last 25 years, ECT-based strategies have been investigated in many preclinical models to assess their potential medical application, mainly in chronic diseases such as type 1 diabetes, hemophilia, cancer, neurodegenerative diseases, lysosomal-storage diseases, and renal failure.^1^, ^2^, ^3^, ^4^, ^5^, ^6^, ^7^, ^8^, ^9^, ^10^, ^11^ In some indications, this technology has already been tested in clinical studies.^12^, ^13^, ^14^, ^15^

The cell encapsulation process involves the immobilization of modified allogeneic cells within a semipermeable and biocompatible membrane that allows the continuous release of a specific therapeutic proteins. While microencapsulation relies on the use of hydrogel microspheres typically made of alginate, macroencapsulation allows the use of larger capsules or devices that can be tailored according to the intended site for protein delivery.^16^ Our work is focused on macrocapsules that can be easily retrieved at the end of therapy or in the case of loss of function or adverse event, which is a major biosafety improvement over microcapsules.^17^ The size-selective permeability of the biocompatible membrane permits the bidirectional diffusion of molecules that are essential for cell survival inside the capsules, such as nutrients and oxygen, as well as the outward release of the metabolic residues and therapeutic molecules produced after capsule implantation in the host organism by the transplanted cells by passive diffusion. The semipermeable membrane also confers a protective barrier that isolates the encapsulated cells from the immune system of the host. However, the level of protection that the encapsulation provides may depend on membrane permeability to large macromolecules mediating humoral immunity in the host, such as antibodies and complement proteins. The use of capsules with large membrane pores may be required to deliver large therapeutic molecules such as antibodies; however, while these pores may contribute to maximizing the amount of therapeutic product released,^18^ they may also allow the host’s own antibodies to enter the capsule and react against the encapsulated cells. Therefore, these cells must be as non-immunogenic as possible to limit an immune reaction in the host.

Indeed, while several groups have reported the survival of xenogeneic cells in various implantation sites,^14^^,^^19^^,^^20^ other studies have shown that encapsulated xenogeneic cells can elicit immune responses in the host that lead to their rejection,^21^, ^22^, ^23^, ^24^ probably due to the presence of xenoantigens that trigger this effect.^25^, ^26^, ^27^ Therefore, the use of a single, well-characterized source of allogeneic human cells with limited immunogenicity may facilitate the standardization of cell macroencapsulation for use in the clinical setting in any patient’s population.

The development of ECT for clinical purposes highly depends on the use of encapsulated cells with specific characteristics. For instance, in macrocapsules, the low surface/volume ratio limits the diffusion of oxygen and nutrients, consequently, genetically modified cells need to survive in an environment with metabolic restrictions inside the capsule while maintaining stable transgene expression over several days or months.

The encapsulation of the murine immortalized C2C12 myoblast cell line has been successful in multiple preclinical studies.^28^, ^29^, ^30^ The choice of this cell type was guided by the fact that muscle progenitors are able to survive and proliferate in hypoxic conditions.^31^ When maximal cell density is achieved within the capsules, oxygen and nutrients may not efficiently reach the cells at the center of the capsule, leading to a necrotic core than can induce immunogenicity in the host.^32^ The ability of C2C12 myoblasts to resist hypoxia alleviates this risk. Indeed, many studies using myoblasts for cell encapsulation have shown that these cells were able to survive for long periods inside the capsule.^29^^,^^32^, ^33^, ^34^, ^35^, ^36^ Interestingly, their survival under restrictive metabolic conditions did not affect protein secretion.^29^^,^^35^ For example, in a mouse model of anemia, all mice implanted with encapsulated C2C12 myoblasts secreting mouse erythropoietin presented an increase of more than 85% in the hematocrit as early as day 7 after implantation, which was sustained for at least 80 days.^29^

In addition to C2C12 myoblasts, other murine or human cell types have been tested in proof-of-concept and exploratory studies; however, to date, no encapsulated cell line has been specifically established for future use with ECT in humans.^21^^,^^37^ To guarantee patient safety, the regulatory framework for the use of allogeneic cell lines in humans is restrictive. Many available common human cell lines used for therapeutic protein production do not qualify because of poor stability, tumorigenicity, or insufficient viral safety. The development of stem cell technologies has brought new opportunities for cell encapsulation and artificial organ. A lot of effort is made to use encapsulated stem cell-derived pancreatic progenitors for the treatment of diabetes.^38^^,^^39^ However, for the delivery of exogenous therapeutic proteins, mesenchymal stem cells have been disappointing so far due to limited viability after encapsulation and implantation in animal models.^40^^,^^41^ Based on preclinical studies using murine myoblasts for cell encapsulation and previous results on neuromuscular disorders showing that human myoblasts can be immortalized,^42^, ^43^, ^44^ we hypothesized that a human myoblast cell line would be the best candidate to develop a cell macroencapsulation program intended for protein delivery in a clinical setting.

Here, we have generated a stable human myoblast cell line that was genetically modified to secrete diverse functional molecules, including a viral protein (SARS-CoV-2 spike protein), a cytokine adjuvant (human granulocyte-macrophage colony-stimulating factor [GM-CSF]) currently in phase I and II clinical trials (NCT02999646 and NCT02193503) and antibodies (anti-CD20, anti-CTLA4). We performed ECT using this new cell line and observed the long-term secretion of the chosen therapeutic proteins. Our results support that the macroencapsulated immortalized human myoblasts used in this study can be an effective platform for the delivery of various therapeutic factors.

## Results

### Primary human myoblast immortalization after lentiviral CDK4 and hTERT transduction

**Figure 1:**
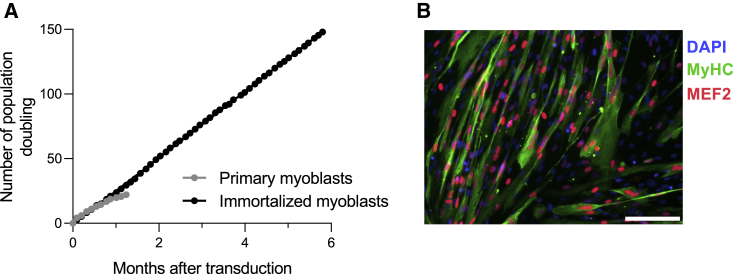
Primary human myoblasts are efficiently immortalized after transduction with lentiviruses encoding for CDK4 and hTERT (A) Primary myoblast cells were transduced with lentiviral vectors encoding the CDK4 and hTERT proteins. Primary and immortalized myoblasts were maintained in culture and proliferation was quantified by determination of population doubling. Each dot represents the mean of two cell counts. (B) Human immortalized myoblasts were expanded and cell differentiation was promoted by removing growth factors from the culture medium. The expression levels of MEF2 and MyHC proteins were quantified by *in vitro* immunofluorescence to assess the myogenic differentiation process. The image is representative of at least seven images from one biologically independent experiment. Scale bar, 50 μm.

The human phosphoglycerate kinase (PGK) promoter contains hypoxia response elements, enhancing transgene expression under hypoxic conditions.^45^ As cell encapsulation may induce suboptimal or hypoxic conditions, we chose the PGK promoter for all vectors used. Using green fluorescent protein-expressing vectors, we confirmed that human PGK-driven expression was increased in human primary myoblasts incubated at 1% O_2_ for 36 h (Figure S1). Next, primary myoblast cells were transduced with lentiviral vectors encoding the cyclin-dependent kinase 4 (CDK4) and human telomerase reverse transcriptase (hTERT) proteins, two factors that were shown to be sufficient to immortalize human myoblast cells while maintaining a differentiation potential and stable karyotype.^46^ Cells were maintained in culture and proliferation was quantified. While primary myoblast cells stopped proliferating after 45 days, cells transduced with CDK4 and hTERT continued growing stably for up to 6 months (Figure 1A). Immortalized myoblasts were differentiated *in vitro* and analyzed by immunofluorescence staining of myocyte enhancer factor-2 (MEF2) and myosin heavy chain (MyHC) proteins to determine their ability to fuse into multi-nucleated myotubes. MEF2 proteins are a family of transcription factors which are important regulators of skeletal muscle differentiation and are expressed only in differentiated cells. MyHC is expressed only in terminally differentiated myotubes.^47^ We observed that most cells fused into myotubes (MyHC-positive cells) expressing MEF2, confirming that transduction of primary myoblasts with lentiviral vectors encoding the CDK4 and hTERT proteins can efficiently immortalize cells without affecting their myogenicity (Figure 1B).

**Figure 2:**
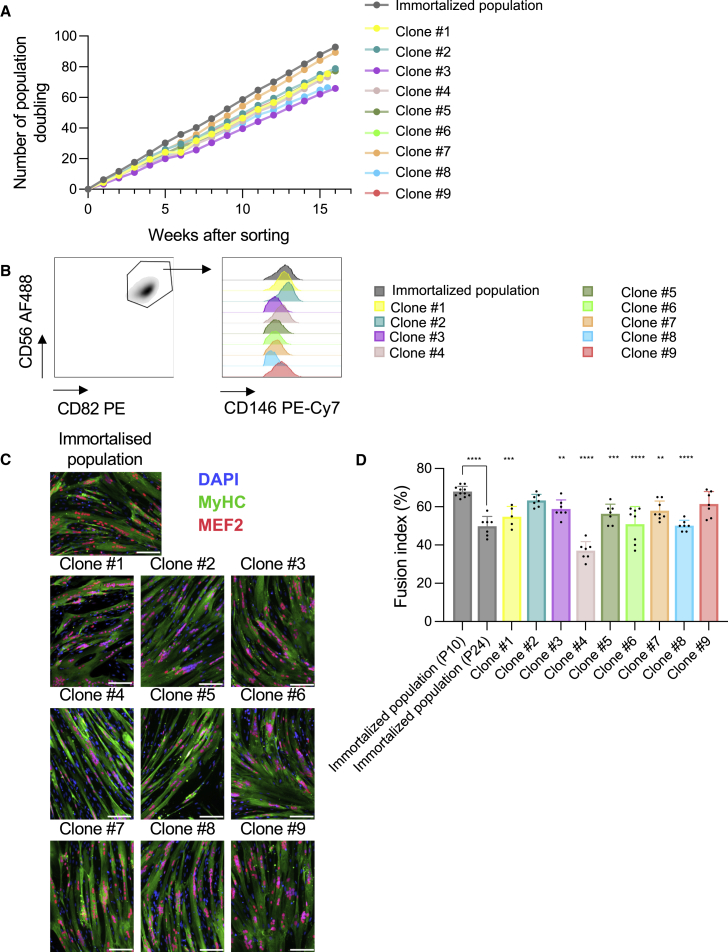
Individual immortalized clones demonstrate preserved myogenic characteristics (A) Individual clones were sorted from the immortalized myoblast cell population using single-cell cloning. The proliferation rate of nine selected clones was quantified by determination of population doubling. Each dot represents the mean of two cell counts. (B) Flow cytometry gating strategy to define the percentage of cells expressing myogenic markers (CD56+ CD82+ CD146+). (C) Individual immortalized clones were expanded and cell differentiation was promoted by removing growth factors from the culture medium. The expression levels of MEF2 and MyHC proteins were quantified by *in vitro* immunofluorescence to assess the myogenic differentiation process. (D) The fusion index was calculated on approximately 200 cells for each individual clone. The immunofluorescence images are representative of at least five images from one biologically independent experiment. Data and errors bars represent mean ± SD from at least five pictures from one independent experiment. The fusion index of each clone was compared with the immortalized population P10 with a one-way ANOVA test. Scale bar, 50 μm.

Individual clones were isolated from the immortalized myoblast cell population by single-cell sorting. We quantified the proliferation rate of nine selected clones and observed that these clones remained stable over time (Figure 2A). To confirm that myoblast cell clones kept their myogenic characteristics after immortalization and sorting, the stability of myogenic marker expression over time was quantified by flow cytometry (triple positivity for CD56, CD82, and CD146) using the gating strategy depicted in Figure 2B. In addition, we measured cell capacity to fuse and differentiate into myotubes (Figures 2C and 2D). Except for clone no. 4, all other clones showed myogenic characteristics with slight variation between them (clone no. 8: 50.14%–clone no. 2: 63.29%). Based on the proliferation rate, differentiation capacity, and survival assessment, we chose clone no. 2 to be transduced with different concentrations of lentivirus for the expression of proteins with therapeutic potential, including human GM-CSF, anti-CD20 IgG, and anti-CTLA-4 IgG, as well as the SARS-CoV-2 spike protein.

### Expression of functional human GM-CSF in immortalized myoblast clones

**Figure 3:**
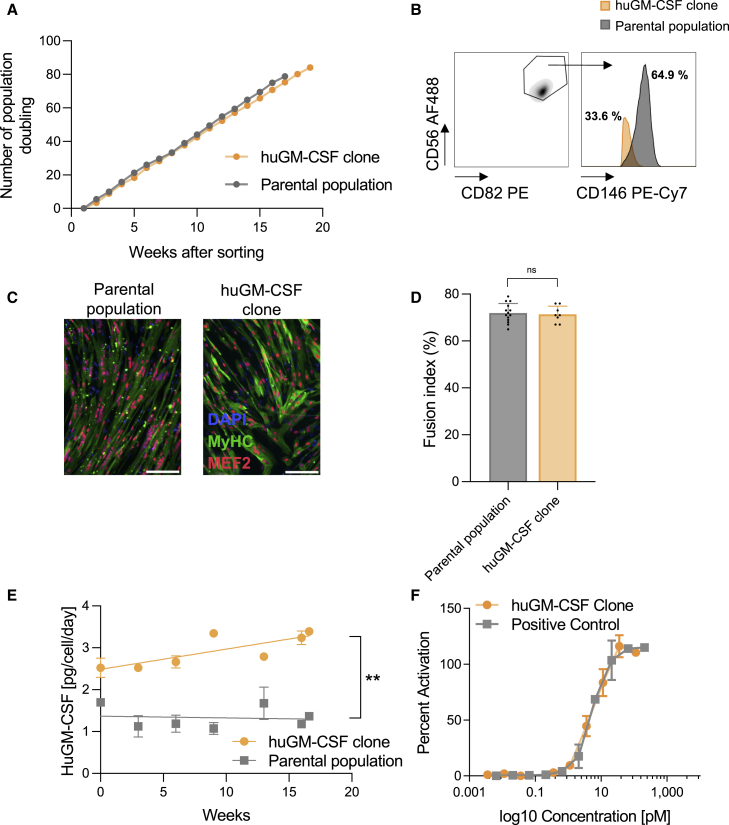
Selected immortalized clones can be efficiently transduced for the expression of functional cytokines: example of human GM-CSF (A) Clone no. 2 (parental population) was transduced with lentivirus for the expression of human GM-CSF. Individual clones were then sorted by flow cytometry single-cell sorting and a clone was selected for its huGM-CSF production. The huGM-CSF clone was maintained in culture and proliferation was quantified by determination of population doubling. Each dot represents the mean of two cell counts. (B) Flow cytometry gating strategy to define the percentage of cells expressing myogenic markers (CD56+ CD82+ CD146+). (C) The huGM-CSF clone was expanded and cell differentiation was promoted by removing growth factors from the culture medium. The expression levels of MEF2 and MyHC proteins were quantified by *in vitro* immunofluorescence to assess the myogenic differentiation process. Scale bar, 50 μm. (D) The fusion index was calculated on around 200 cells. The immunofluorescence images are representative of at least five images from one biologically independent experiment. Data and errors bars represent mean ± SD from at least seven pictures from one independent experiment. The fusion index was compared by a two-tailed unpaired t test. (E) The huGM-CSF secretion by the parental population or by the huGM-CSF clone was quantified by ELISA. Data and error bars represent mean ± SD of n = 2. Lines represent a linear regression, two-way ANOVA with repeated measure. For group effect p = 0.027. No significant effect of time (p = 0.0732). (F) The potency assay for the secreted GM-CSF was evaluated by the LanthaScreen STAT5 TF-1 cellular assay (Thermo Fisher Scientific). Potency capacity was directly correlated with the half maximal effective concentration (EC_50_) and was determined with a 10-point titration curve. Recombinant GM-CSF was used as a positive control. Data and error bars represent mean ± SD of two experiments.

To assess the ability of immortalized myoblast clones to express functional human GM-CSF, clone no. 2 was transduced with different lentiviral multiplicity of infection (MOI) driving the expression of this protein. The secretion of human GM-CSF from cell populations generated from transduced immortalized clone no. 2 was monitored weekly for 9 weeks, remaining stable at levels depending on the MOI (Figure S2). Cells were able to secrete 0.15 ± 0.018, 0.43 ± 0.065, and 1.33 ± 0.26 pg/cell/day after transduction with an MOI of 3, 10, and 100, respectively. We then performed single-cell cloning. Minimal differences were observed among clones and one of them was further characterized with the objective of using this clone for clinical testing. The proliferation rate of the selected GM-CSF-secreting clone (huGM-CSF clone) was monitored for several months and compared with the parental population (clone no. 2 transduced with MOI = 100). The population doubling of the selected human GM-CSF clone increased in a stable and constant way, similar to what was observed in the parental cell population (Figure 3A), suggesting that transduction with human GM-CSF did not affect proliferation capacity.

Again, to confirm that the selected huGM-CSF clone kept its myogenic characteristics after transduction and sorting, the stability of the expression of myogenic markers was quantified by flow cytometry (triple positivity for CD56, CD82, and CD146) and the capacity to fuse and differentiate into myotubes was measured. Although expression of myogenic markers was lower than in the parental cell population (Figure 3B), the capacity to differentiate into myotubes (Figure 3C), measured by the fusion index, was unchanged (Figure 3D) (parental cell population 71.93% ± 4.066% versus huGM-CSF clone 71.38% ± 3.503%, p = 0.7157). Finally, human GM-CSF secretion by the huGM-CSF clone was monitored for several months (Figure 3E). We observed that human GM-CSF secretion in the parental cell population varied between 1.075 (min) and 1.697 (max), with a mean of 1.330 pg/cell/day for 16 weeks, whereas secretion in the huGM-CSF clone had a mean of 2.973 pg/cell/day (min: 2.524 pg/cell/day; max: 3.452 pg/cell/day). In both cell populations, secretion remained stable over time. In addition, the functionality of the GM-CSF secreted from the immortalized myoblast clone was tested using the LanthaScreen STAT5 TF-1 potency cellular assay.^48^ The half-maximal effective concentration (EC_50_) for secreted GM-CSF was equivalent to that of a commercially available cytokine (0.00527 versus 0.0054 pM, Zʹ = 0.6 (Figure 3F). This confirmed that immortalized human myoblasts can efficiently be transduced to produce highly stable functional cytokines, such as human GM-CSF.

### Delivery of a huGM-CSF using encapsulated human myoblasts

**Figure 4:**
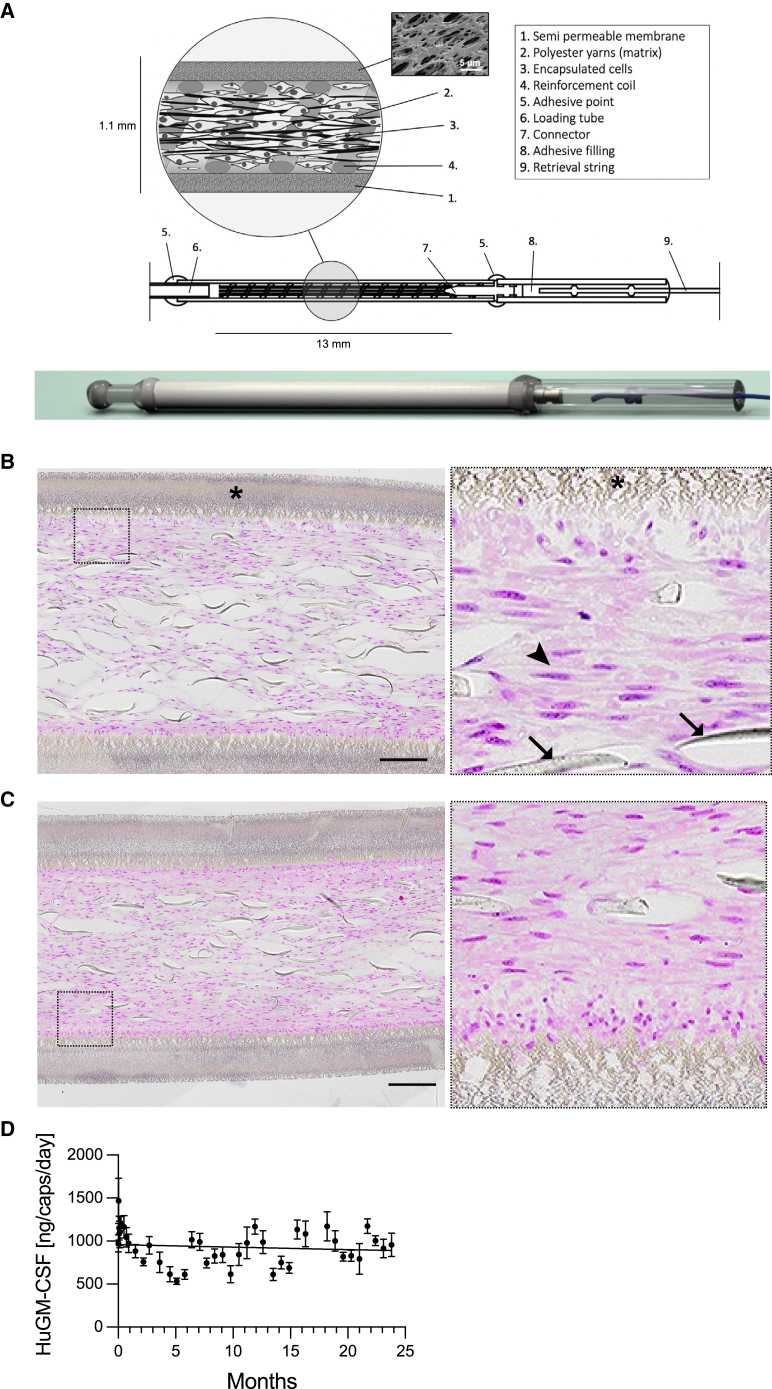
*In vitro* evaluation of encapsulated myoblasts as a delivery system (A) Scheme of the MyoPod capsule depicting the various device components. The huGM-CSF-secreting myoblast clone was encapsulated and kept in culture. Capsules were fixed and processed for histological analysis. Qualitative assessment of survival was then performed by hematoxylin and eosin staining. (B) After 1 week *in vitro* viable cells were organized in a tissue with aligned nuclei (the right panel is the magnified delimited area). Arrowhead shows aligned nuclei. Arrows point to the yarn that is used as a supporting matrix. Asterisks show the semipermeable membrane. (C) After 3 weeks *in vitro*, similar observations were made, suggesting a sustained cell survival over time. (D) The huGM-CSF clone was encapsulated and kept in culture for up to 2 years. Regularly (each day during the first week, each 2 weeks, and then each 3 weeks), a 2-h release assessment was performed to evaluate huGM-CSF secretion quantified by ELISA. This experiment used n = 7 capsules. The line represents a linear regression with slope not different from zero, p = 0.0996.

For the immortalized myoblast, we engineered a companion semipermeable macroencapsulation device (the MyoPod medical device), which is schematized in Figure 4A. This device was specially designed for the encapsulation of adherent cells and implantation in the subcutaneous tissue. It is composed of a hollow fiber semipermeable membrane made of modified polyether sulfone. The cylindrical chamber containing encapsulated cells has a volume of 5 μL. The hollow fiber structure of the device facilitates the implantation in the target tissue through a trocar. We included polyester yarn as a supporting matrix for adherent cells. Cells are loaded on one end through a loading tube that is sectioned and sealed before implantation. On the other end, an anchoring tube is included to manipulate the device during manufacturing without damaging the semipermeable membrane and tethering a thread that is used for the retrieval of the device. All the elements of the device are assembled using a medical-grade UV-curing adhesive. The device can be produced under GMP conditions and went through chemical characterization, toxicological assessment, and biocompatibility testing according to the EN ISO 10993 standards.

Cells from the huGM-CSF clone and clone no. 2 were encapsulated in the MyoPod encapsulation device and were kept in culture to assess stability of expression and survival inside the device. Histological analysis was performed after 1 and 3 weeks *in vitro*. Already after 1 week *in vitro*, viable cells from the huGM-CSF clone were forming a dense organized tissue that spread out through the entire chamber of the capsule (Figure 4B). Higher magnification shows typical alignment of muscle cell nuclei along the axis of the supporting matrix. After 3 weeks *in vitro*, the findings were consistent (Figure 4C). Similar results were obtained for the immortalized clone (Figure S3). The GM-CSF secretion rate in the culture medium was assessed for 2 years in a long-term stability study (Figure 4D). Despite some variability, the overall yield of secretion in medium remained stable over time at about 1 μg/day, suggesting that encapsulated cells reached an equilibrium inside the device (Figure 4D).

**Figure 5:**
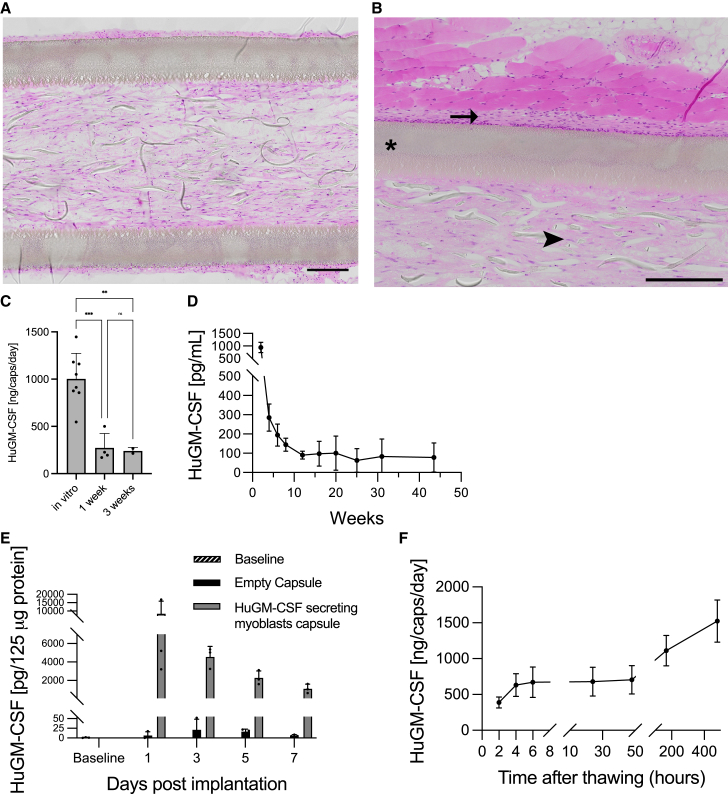
*In vivo* delivery of huGM-CSF The huGM-CSF-secreting clone was encapsulated. Capsules were implanted into Rag2/IL2rg^−/−^ mice and kept for 1 or 3 weeks in the mice. (A) Histological section of the device after 1 week implantation in the mouse subcutaneous tissue. Hematoxylin and eosin staining demonstrates cell survival inside the MyoPod device. (B) After 3 weeks *in vivo*, numerous viable myoblastic cells are observed in the inner device chamber (arrowhead). A limited cellular infiltrate is observed in the surrounding subcutaneous tissue (arrow). The asterisk depicts the semipermeable membrane. (C) Capsules were then explanted and, the day after the explantation, a 2-h release was performed to evaluate huGM-CSF secretion quantified by ELISA. One-way ANOVA of n = 2–6 replicates. (D) Serum samples were isolated from implanted mice to quantify huGM-CSF levels. N = 4-5. (E) Evaluation of huGM-CSF secretion by encapsulated cells implanted in an *ex vivo* human skin model. Each bar represents mean ± SD of n = 3 replicates per time point per condition. (F) Capsules were frozen into liquid nitrogen and, after thawing, huGM-CSF secretion was evaluated by ELISA. Each dot represents mean ± SD of n = 72 replicates.

Next, in an attempt to predict the performance of encapsulated huGM-CSF in patients, we used two different models. First, we implanted capsules in the subcutaneous tissue of immunodeficient Rag2/IL2rg^−/−^ mice. An immunocompromised model was selected to minimize the immune response against the xenogenic component of the engineered transplanted cells and avoid a neutralizing anti-drug response that may alter the blood level of delivered recombinant protein. This mouse model does not develop functional T, B, and NK cells, which results in deficient adaptive immune response. Here, we aimed at testing the capacity of the novel cell line to cope with metabolic and local constrains inside the encapsulation device. After 1 and 3 weeks *in vivo*, devices were explanted, huGM-CSF secretion was quantified, and capsules were fixed and processed for histology. Histological analysis revealed encapsulated cell viability similar to *in vitro* conditions. No major necrotic core was observed and viable cells were detected throughout the entire capsules (ID = 750 μm) (Figure 5A). After 3 weeks *in vivo*, a limited cell infiltrate was found in the outer part of the membrane, but no significant fibrotic reaction was found in the surrounding tissue (Figure 5B). The secretion of huGM-CSF was quantified from explanted devices. Compared with *in vitro* capsules, *in vivo*-implanted capsules presented a decrease in secretion. Following retrieval, the average secretion rate from the capsules was 27.25% of the pre-implantation rate after 1 week *in vivo* and 23.96% after 3 weeks *in vivo*. No difference in the secretion level was observed between capsules that were implanted for 1 or 3 weeks (Figure 5C). Seric huGM-CSF was detected in implanted mice (Figure 5D). Although it reached a maximum of 932.93 ± 206.72 pg/mL immediately after implantation, seric huGM-CSF decreased during the first 12 weeks and then stabilized at around 80 pg/mL for more than 10 months. To further characterize the delivery of human GM-CSF in subcutaneous tissue from encapsulated myoblasts, an *ex vivo* human skin model was used (HypoSkin). We were able to detect huGM-CSF in the skin surrounding the capsules. A decrease in the environmental GM-CSF concentration was observed from day 1 after implantation until day 7 after implantation. The average quantity of huGM-CSF in the tissue 1 day after implantation was 8,457.25 ± 7,428.24 pg/125 μg protein; after this, the average was 4,550.63 ± 1,145.5 pg/125 μg protein, 2,278.63 ± 764.85 pg/125 μg protein, and 1,099.76 ± 495.37 pg/125 μg protein after 3, 5, and 7 days, respectively. No reactive endogenous GM-CSF secretion was measured with the implanted empty capsule (Figure 5E).

### Secretion of GM-CSF from encapsulated human myoblasts after freezing and thawing

Capsules containing GM-CSF myoblasts were frozen in liquid nitrogen, then thawed and maintained in culture. Human GM-CSF secretion was then quantified by ELISA at various time points (Figure 5F). During the first 2 h after thawing, the encapsulated huGM-CSF clone secreted 389.72 ± 76.65 ng/caps/day. Four hours after thawing, secretion had increased to 632.75 ± 160.11 ng/caps/day and remained stable during more than 48 h. After this time, secretion further increased to 1,523.89 ± 293.64 ng/caps/day at 20 days after thawing.

### Chronic delivery of therapeutic macromolecules with preserved functionality from human myoblasts

We then tested the capacity of the newly derived immortalized myoblast population to deliver complex macromolecules. For these proof-of-concept experiments, we chose two antibodies that are currently used as therapeutic agents in medical oncology, rituximab (Rituxan, a commercialized therapeutic anti-CD20 monoclonal antibody created according to methods described previously^49^) and ipilimumab (Yervoy, anti-CTLA-4, an immune checkpoint inhibitor).

**Figure 6:**
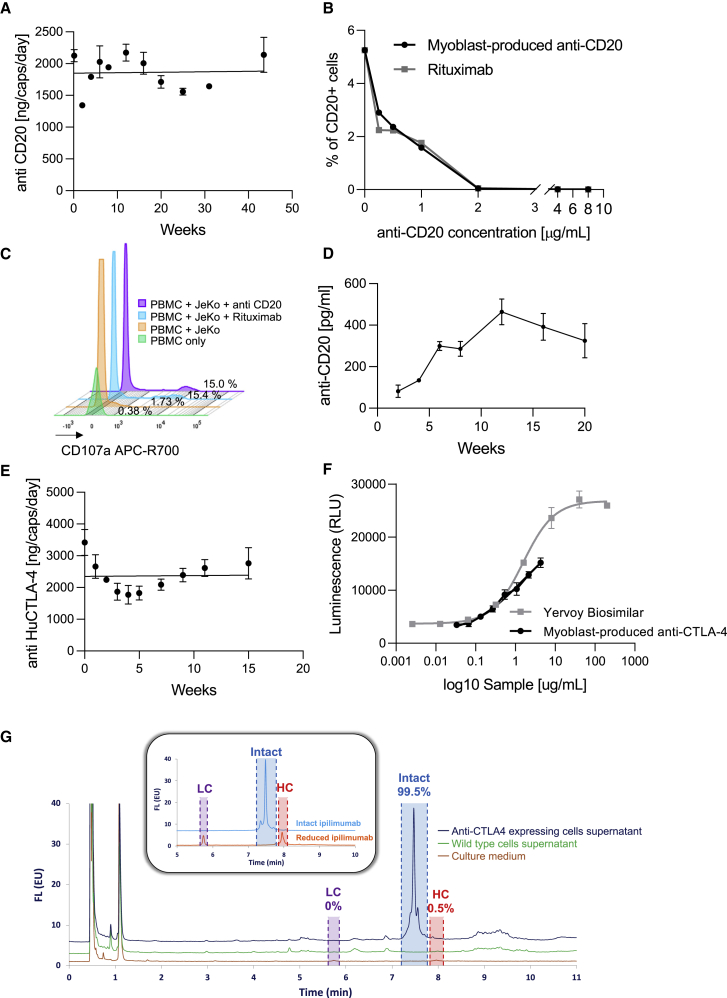
Novel immortalized myoblasts can be used for the continuous delivery of complex therapeutic macromolecules with preserved functionality (A) Human anti-CD20-expressing myoblasts were encapsulated and kept in culture for up to 40 weeks. Repeated quantification of anti-CD20 levels was performed by ELISA during the course of the experiment. Each point represents the mean of two technical replicates. The line represents a linear regression with a slope not different from zero, p = 0.7246. (B) The specific binding between human anti-CD20 produced by myoblasts and human CD20 was evaluated by a flow cytometry competition assay. Human PBMCs were pre-incubated with different concentrations of either the myoblast-produced anti-CD20 IgG or commercial rituximab and then incubated with a fluorescent-tagged anti-CD20 antibody. The percentage of cells expressing CD20 was then assessed. (C) The functionality of human anti-CD20 produced by myoblasts was evaluated by an antibody-dependent cellular cytotoxicity (ADCC) assay. A mantle cell lymphoma (MCL) cell line expressing CD20 (JeKo-1) pre-exposed to either rituximab or myoblast-produced anti-CD20 were co-cultured with PBMCs for 4 h. The upregulation of the degranulation marker CD107a expression was evaluated on NK cells (CD56+ CD3^−^, CD14^−^ CD19^−^) by flow cytometry. (D) The anti-CD20-secreting myoblast cells were encapsulated, implanted into Rag2/IL2rg^−/−^ mice, and kept for 5 months in the mice. Human anti-CD20 was quantified in the mouse serum over the course of the experiment. N = 5. (E) Human anti-CTLA-4-expressing myoblasts were encapsulated and kept in culture for 15 weeks. Repeated quantification of anti-CTLA-4 levels was performed by ELISA during the course of the experiment. Each point represents the mean of six technical replicates. The line represents linear regression with a slope not different from zero, p = 0.8791. (F) The CTLA-4 blockade assay was performed using myoblast-produced supernatant containing anti-CTLA-4. The blockade capacity was directly correlated with the EC_50_ and determined with an eight-point titration curve. Each point represents the mean ± SD of two technical replicates. (G) RPLC-FL chromatograms of intact commercial ipilimumab and reduced ipilimumab (inset), as well as culture medium, supernatant from wild-type immortalized myoblast cells, supernatant for anti-CTLA4-expressing myoblasts. Time windows for peaks integration were highlighted in purple, red, and blue. Analytical conditions are described in the materials and methods.

First, the immortalized clone no. 2 was transduced with separate lentiviral vectors encoding for either the heavy chain (HC) or light chain (LC) of rituximab to generate stable antibody-expressing cells. The use of separate vectors had been shown to be an efficient method for the stable expression of antibodies by encapsulated cells.^49^ Stably transduced myoblasts produced 14.23 ± 3.36 pg/cell/day. After encapsulation in MyoPod device, the system could deliver 1,860.45 ± 270.92 pg/capsule/day for at least 10 months *in vitro* (Figure 6A). We then studied the functionality of the produced anti-CD20 antibody using a flow cytometry competition assay. We assessed the specific binding between myoblast-produced anti-human CD20 in supernatant and human CD20. Human peripheral blood mononuclear cells (PBMCs) were pre-incubated with different concentrations of either myoblast-produced anti-CD20 or commercial rituximab and then incubated with a fluorescent-tagged anti-CD20 antibody. We found that the produced IgG behaved exactly like rituximab in this assay (Figure 6B). Moreover, the myoblast-produced anti-CD20 was as efficient in a degranulation assay that tested the induction of a cytotoxic response (overexpression of CD107a) after exposition of a B cell line (Figure 6C). The capacity of our new encapsulation system to chronically deliver a therapeutic antibody was then tested in an *in vivo* experiment. Anti-CD20 secreting myoblasts were encapsulated in the MyoPod device and implanted in the subcutaneous tissue of Rag2/IL2rg^−/−^ mice for 5 months. The plasma concentration of anti-CD20 peaked at 462.8 ± 139.3 pg/mL on week 12 (Figure 6D).

We then used a similar approach to evaluate the capacity of the human myoblast cells to secrete ipilimumab, an immune checkpoint inhibitor that is associated with systemic toxicity that could potentially be decreased by a local controlled delivery.^50^ The immortalized clone no. 2 was transduced as described previously. After transduction, cells produced in culture 4.18 ± 0.31 pg/cell/day. Following encapsulation in the MyoPod device, 2,364.53 ± 566.84 pg/capsule/day could be delivered for at least 3 months (Figure 6E). The antibody functionality was assessed by blockade activity using a commercially available kit based on the interaction between samples or control samples with cytotoxic T lymphocyte-associated protein 4 (CTLA-4), which would block the interaction between CTLA-4 and CD80/86. This activity was compared with a Yervoy biosimilar antibody. Myoblast-secreted anti-CTLA4 in culture supernatant displayed significant blockade activity against the interaction between CTLA-4 and CD80/86 (Figure 6F). We used growth medium as a negative control and observed that it did not block the interaction between CTLA-4 and CD80/86. The EC_50_ from the positive control and myoblast-secreted anti-CTLA4 were similar (1.573 versus 1.519, respectively), confirming a normal activity for the IgG secreted by the modified cells. From a regulatory standpoint, the purity of secreted antibody had to be demonstrated. Because a dual lentivector strategy had been used for HC and LC transduction, we performed a chromatography analysis to exclude the secretion of non-functional free HC or LC. Various samples were analyzed using reversed-phase liquid chromatography with fluorescence (RPLC-FL) as described in the materials and methods, namely intact ipilimumab (from commercial origin), reduced ipilimumab, culture medium, supernatant from immortalized myoblast cells (not expressing mAb), and supernatant from anti-CTLA4-expressing myoblasts. Figure 6G shows the chromatograms corresponding to these five different samples. As illustrated in the inset of Figure 6G, the peaks corresponding to intact ipilimumab, LC, and HC were well separated by the developed RPLC method based on their hydrophobicity, and the following time windows were considered for peak integration/quantitation: LC eluted between 5.6 and 5.9 min (purple dotted lines), HC eluted between 7.8 and 8.1 min (red dotted lines), and intact ipilimumab eluted between 7.15 and 7.75 min (blue dotted lines). The chromatograms corresponding to culture medium, culture medium with cells (not expressing mAb), and supernatant from anti-CTLA4-expressing myoblasts were also reported. First, it is important to notice that the FL conditions employed in this work are specific for tryptophan and protein species. As confirmed by the brown trace, the peaks eluted before 2 min correspond to amino acids contained in the culture medium. The cells are also responsible for additional peaks on the chromatograms (illustrated by the green trace) that should correspond to protein species, but these peaks were eluted outside the LC, HC, and intact mAb time windows. Finally, the dark blue trace allows direct quantification of the amount of LC, HC, and intact mAb, based on the corresponding peak areas for the time windows previously mentioned. As calculated, the supernatant from anti-CTLA4-expressing myoblasts contained more than 99% of intact anti-CTLA4. A full characterization of various peaks in reducing and non-reducing conditions is presented in Figure S4 and Table S1.

Finally, immortalized myoblast cells were modified to secrete a modified trimeric version of the SARS-CoV-2 spike protein missing the transmembrane domain.^51^ The supernatants from cultured cells and encapsulated cells were transferred to a PVDF membrane and developed after incubation with a characterized primary antibody.^52^ We detected the spike protein in both cell and capsule supernatants (Figure S5).

## Discussion

In this study, we generated an immortalized human myoblast cell line specially designed for the delivery of therapeutic proteins using macroencapsulation. ECT has been proposed for various clinical applications including chronic drug delivery for hormone replacement therapy,^1^, ^2^, ^3^ treatment of neurodegenerative disorders,^14^ and intraocular delivery.^12^ This technology relies on the capacity of the capsule’s semipermeable membrane to isolate the grafted allogeneic cells from the host’s immune system. One of the major determinants that may prevent the successful engraftment and sustained survival of encapsulated cells is the formation of an initial dense necrotic core that releases intracellular antigens from encapsulated cells, resulting in a sensitization of the host immune system.^32^ This core emerges from the imbalance between cellular metabolic needs and the transport of nutrients and oxygen to the center of the device,^53^ and is promoted by over-proliferation of the grafted cells.^32^ Interestingly, if the initial cell death can be prevented, for instance, by decreasing cell density or using potent cell lines, the host immune response is limited and the mechanical barrier between grafted cells and host cells is sufficient to prevent the rejection of allogeneic cells.^32^ This remains true even if the membrane pore size allows for the diffusion of large molecules such as antibodies.^25^

Despite many interesting preclinical studies, the clinical development of ECT using macrocapsules remains limited, partly because of the lack of compatible human cell lines to use with this technology. Indeed, there are currently no human cell lines for macroencapsulation delivery that have reached market approval. A substantial amount of clinical data has been produced for encapsulated pancreatic islets (reviewed in Santos-Vizcaino and co-workers^17^^,^^54^), but very few genetically modified human cell lines have successfully entered into early-stage clinical trials. The ARPE-19 cells derived from retinal pigment epithelia are currently used in phase II and III clinical trials but only for intravitreal implantation, limiting its range of potential therapeutic applications^55^ (NCT03319849, NCT02862938). They were also used in phase I for the intracerebral delivery of nerve growth factor in Alzheimer’s disease and modified for the delivery of glial cell line-derived neurotrophic factor in Parkinson’s disease.^56^^,^^57^ While this cell line has demonstrated sustained survival inside macrocapsules, the yield of stable protein delivery after transduction remained limited (below 1 pg/cell/day) even after optimizing the transduction conditions using a transposon.^58^^,^^59^

Here, we have produced an immortalized human myoblast cell line that was designed and selected for its compatibility with encapsulation systems. Similar to the C2C12 mouse myoblasts that have been extensively used in preclinical studies, our novel cell line was able to resist the metabolic restrictions inside the hollow fiber capsules without forming any significant necrotic core.

In immuno-oncology, GM-CSF has been proposed as a potent immunostimulatory adjuvant for vaccination against cancer cells; it was shown to recruit and activate dendritic cells and thus promotes the development of antigen-specific T cells.^60^ However, the administration of systemic or higher doses of GM-CSF has been associated with the expansion of myeloid-derived suppressor cells that have a deleterious immunoinhibitory effect in cancer immunization (reviewed in Parmiani et al.^61^). The local subcutaneous delivery of the cytokine adjuvant GM-CSF by encapsulated cells, together with irradiated autologous tumor cells, has been proposed for cancer immunotherapy and is currently under clinical evaluation (NCT02193503).^62^ Here, we demonstrate that immortalized human myoblasts can be modified to stably express functionally active GM-CSF. When encapsulated, GM-CSF-secreting myoblasts were able to produce this protein for more than 2 years *in vitro*. In mice, in which human GM-CSF is not biologically active, serum levels peaked immediately after implantation, reached a steady state after 10 weeks, and was detected up to 10 months after implantation. Considering the very short half-life of human GM-CSF in mice (6–8 h^63^), the detection of steady GM-CSF serum levels confirms that implanted capsules continuously delivered the adjuvant cytokine at meaningful levels. Interestingly, in an *ex vivo* human skin model, the concentration of GM-CSF in the surrounding tissue decreased during the first week, suggesting that it may induce a strong cellular response and be consumed by attracted cells, as observed previously.^62^

As a proof-of-concept experiment for other potential clinical applications, we demonstrated that the novel myoblast cell line can produce more complex macromolecules, such as the monoclonal anti-CD20 and anti-CTLA4 antibodies, at significant levels. The functionality of both secreted IgGs was verified in specific assays. In a chronic experiment, we have shown that the human encapsulated myoblasts implanted in the mouse subcutaneous tissue, could chronically deliver an anti-CD20 therapeutic antibody for up to 5 months. Our results suggest that the function of the secreted molecule matters, and will impact the scope and the duration of the intended application: while the local delivery of inflammatory molecules should be limited to several days, the delivery of molecules with longer half-life and distant target could be extended to several months. In both cases, further investigations in disease models are required. The combination of modified myoblasts with high-capacity, flat sheet-type subcutaneous capsules may represent a very interesting approach for long-term delivery of therapeutic monoclonal antibodies.^28^^,^^34^

We have also shown that the novel encapsulated cell line can deliver the SARS-CoV-2 viral spike protein, which could be tested for immunization protocols.

The translation of ECT from preclinical to clinical evaluation requires the challenge of manufacturing and supplying an advanced therapy medicinal product to be addressed. The finding that encapsulated immortalized human myoblasts can be frozen (inside the macrocapsule), cryopreserved, and thawed without impairing the delivery of the secreted proteins represents a major advantage for future clinical developments.

Despite the mechanical barrier provided by the macrocapsule membrane between the host and transplanted cells, the immunogenicity of the cell line could potentially prevent its long-term survival in absence of immunosuppressive drug. In mice, the macroencapsulation of the C2C12 myoblast line in allogeneic conditions has produced the most robust results over the years, suggesting that myoblasts have limited immunogenicity when encapsulated. We hypothesize that this feature might be conserved in human myoblasts. Along those lines, previous work has shown that human myoblasts isolated using a similar protocol exhibit immunomodulatory properties that are similar to human mesenchymal stromal cells.^64^ To minimize immunogenicity, myoblast cells were engineered with lentivectors, which are known to generate limited immune or inflammatory reactions by themselves (reviewed in Vargas et al.^65^).

Biosafety is another major concern with cell line-derived therapeutic products. Third-generation lentiviral vectors were demonstrated to be a safe approach for cell line engineering.^66^ Indeed, several therapeutic products (such as chimeric antigen receptor-T cells for leukemia or treatment for β-thalassemia) have already reached phase III clinical trial or market approval and use *ex vivo* cell engineering or *ex vivo* gene therapy with lentivectors with no associated limitation (reviewed in Bulcha and co-workers^67^^,^^68^). Therefore, lentivector-mediated cell engineering should not impact the use of this novel myoblast cell line in humans. The use of an immortalized cell line may raise concerns regarding the risk of carcinogenicity that may prevent advancement to clinical development. Nevertheless, several immortalized cell lines have been clinically tested in immuno-oncology, including the K562 line, a human erythroleukemic cell line that served as the backbone of multiple clinical trials over the last decades without safety concerns.^69^^,^^70^ In addition, the reported cell line is intended to be solely used with macroencapsulation technology, which is a semipermeable macrocapsule. The cells never come into contact with the patient, and the implant can be extracted from the body at all time. The intervention being totally reversible mitigates any carcinogenicity risk to a minimal level. Still, it is expected that the safety of any biotechnology product mediated by viral contamination should be guaranteed.^71^ The GM-CSF-secreting cell line described here was successfully characterized following the ICH Q5 A guidelines and technical requirements, along with compendial analytical methods. Identity, sterility, mycoplasma, viability, human viruses, bovine viruses, viral contaminants, adventitious agents, and vector infectivity were assessed according to compendial methods, and complied with all acceptance criteria (summarized in Table S2). The combination of the GM-CSF-secreting myoblast line and the MyoPod encapsulation device will be evaluated in a multicenter open-label, single arm, phase I clinical trial in Switzerland, in participants with advanced solid tumor (NCT05071846). Subjects will receive six vaccinations consisting in the subcutaneous implantation of encapsulated GM-CSF-secreting myoblast cells and local injection of irradiated autologous tumor cells. This clinical study will integrate secondary endpoints related to pharmacokinetics and pharmacodynamics, such as the analysis of tissue response around the encapsulated devices, which will be very informative.

Together, our results suggest that the novel immortalized myoblast cell line is a robust cell line that can be used in macroencapsulation. We believe that this cell line may support the clinical development of autologous anti-tumor immunotherapy using encapsulated GM-CSF-secreting cells, thereby emerging as a potential candidate for long-term drug delivery. Our work fills an existing gap and opens novel perspectives for the clinical development of other ECT-based therapies.

## Materials and methods

### Cell source and informed consent

Healthy human muscle tissue was obtained from a non-smoking 32-year-old female donor with no significant medical history or chronic disease, who had a reconstruction surgery after a knee traumatism with anterior cruciate ligament injury. The donor provided informed consent for the use of small muscle debris dissected during the surgery (semi-tendinous muscle).

The eligibility criteria for tissue donation were defined according to the Food and Drug Administration guidelines “Eligibility Determination for Donors of Human Cells, Tissues, and Cellular and Tissue-Based Products (HCT/Ps).” The criteria determined that the donor had to be a healthy subject aged more than 18 years and with no identified chronic condition, including muscle disease, who had a planned orthopedic surgery and presented negative screening tests for human immunodeficiency virus types 1 and 2, hepatitis B virus, hepatitis C virus, *Treponema pallidum*, human T cell leukemia virus types 1 and 2, and West Nile virus.

All procedures performed involving this participant were in accordance with the institutional ethical standards, with the Swiss Human Research Act, and with the 1964 Helsinki Declaration and its later amendments or comparable ethical standards. The Cantonal Ethics Committee in Geneva approved the study protocol (BP_2016-01793).

During surgery, the muscle tissue sample (<5 mm) was placed into an empty sterile container and kept at 4°C for less than 2 h until processing and dissociation. The quality of the sample was assessed through the dissociation and flow cytometry protocols described below.

### Isolation of human primary myoblasts

Human muscle tissue sample was rinsed with Dulbecco’s modified Eagle’s medium high glucose (DMEM GlutaMAX Supplement, Gibco, 61965026) in sterile conditions, and fat cells and fibrotic tissue were removed. Muscle tissue was then broken down in a Petri dish containing 5 mL of a Trypsin-EDTA 0.05% solution (Gibco, 25300054), transferred to a sterile bottle containing 90 mL of the same solution and incubated at 37°C for 60 min under agitation for further cell separation. Trypsin action was stopped with 10% fetal bovine serum (FBS) (Gibco, 10101145). The resultant cell solution was then filtered with a 70-μm cell strainer and centrifuged at 1,000 rpm for 5 min at room temperature (RT). The supernatant was discarded and the pellet was resuspended in DMEM and filtered with a 40-μm cell strainer. Cells were plated in myoblast growth medium (GM) prepared with Ham’s F-10 Nutrient Mix GlutaMAX Supplement (Gibco, 41550021) and supplemented with 15% FBS, 0.5 mg/mL bovine serum albumin (BSA) (Sigma Aldrich, A4503), 0.5 mg/mL fetuin (Desert Biological, 302070), 10 ng/mL epidermal growth factor (R&D Systems, 236-GMP-200), 0.39 μg/mL dexamethasone (PharmaServ, 8016), 0.04 mg/mL insulin (Sigma Aldrich, I9278), 149 μg/mL creatine monohydrate (PharmaServ, 8114), 100 μg/mL pyruvate (Gibco, 11360039), 50 μg/mL uridine (Sigma Aldrich, U3003) and 5 μg/mL gentamycin (Gibco, 15710049). Cell cultures were plated at a density of 2 × 10^5^ cells/mL in a 60 mm Petri dish containing 3.5 mL of GM. Cell cultures were maintained in a humidified incubator with 5% CO_2_ at 37°C until cell confluency reached 75%.

Myoblasts were isolated from other cells by flow cytometry cell sorting and were defined as cells expressing CD56+, CD146+, and CD82+. Conditioned GM was removed, and cells were washed with Hank’s balanced salt solution (HBSS) (with no calcium, magnesium, or phenol red; Gibco, 14175053). Using the same Trypsin-EDTA solution mentioned above, cells were detached from the Petri dishes until this reaction was stopped with fresh GM. Cells were then collected and centrifuged at 1,000 rpm for 5 min at RT. The supernatant was discarded, and the pellet was washed with fresh GM before a second centrifugation was performed. Cells were stained for 30 min at 4°C with anti-CD56 Alexa Fluor 488 (BD Biosciences, 557699), anti-CD82 PE (BioLegend, 342104), and anti-CD146 PE-Cy7 (BD Biosciences, 562135). Cells were washed once more and sorted using a MoFlo Astrios EQ flow cytometer (Beckman Coulter) in a 96-well plate containing one cell per well. The cell population doubling at each passage was defined as log(N)/log(2), where N is the number of cells collected divided by the number of cells initially seeded. Individual clones were then allowed to grow and expand in culture.

### Cell transduction with lentiviral vectors

For cell transduction experiments, myoblasts were cultured at a density ranging from 2 × 10^4^ to 3 × 10^4^ cells/mL in a 24-well plate with 500 μL of GM per well and incubated as described above.

The cDNAs for the catalytic subunit of the hTERT, CDK4, and GM-CSF were provided by Geneva University’s Vectorlab platform and subcloned into a third-generation pCLX lentiviral vector backbone (Addgene, 45956) under the expression of the human PGK promoter.^31^^,^^32^^,^^72^ Lentiviral particles encoding hTERT, CDK4, and GM-CSF were produced by co-transfection of the pCLX, pMD2.G (Addgene, 12259), and psPAX2 plasmids (Addgene, 12260), as described elsewhere.^72^ For expression of the SARS-CoV-2 spike protein, the 1–1,208 amino acids were used in the construct. Transmembrane and endoplasmic reticulum retention domains were removed from the original sequence. A T4 fibritin trimerization domain was included, two prolines were substituted at residues 986 and 987 to stabilize the trimeric structure, and the GSAS motif was substituted at the furine cleavage site 682–685.^51^ Lentiviral particles for the expression of the other proteins were ordered from Vectorbuilder. The specific viral infectivity of each vector was titrated on HT-1080 cells and expressed in transducing units per mL.^73^

Cell transduction with lentiviral particles was performed 1 day after the myoblast culture was prepared. In brief, 250 μL of conditioned GM solution containing 5 μg/mL polybrene and viral particles were added to the myoblasts. Lentiviral particles presented a MOI ranging from 3 to 30 for each vector. Cultures were then incubated for 24 h at 37°C. The following day, 500 μL of fresh GM was added to each well and, 24 h later, cells were transferred to a new culture dish.

### Myotube differentiation and immunofluorescence

The myogenic potential of the isolated myoblasts and myotube differentiation were assessed by immunofluorescence analysis of the MEF2 and MyHC proteins, two markers of myotube formation. Myoblasts were plated on poly-D-lysine-coated coverslips at a density of 2 × 10^5^ cells/mL in 35-mm culture dishes containing 2 mL GM and incubated as described above until cultures reached 100% confluency. Conditioned GM was removed, cells were washed with HBSS and incubated in differentiation medium prepared with DMEM GlutaMAX Supplement and supplemented with 0.5 mg/mL BSA, 10 ng/mL epidermal growth factor, 10 μg/mL insulin, 149 μg/mL creatine monohydrate, 100 μg/mL pyruvate, 50 μg/mL uridine, and 10 μg/mL gentamycin. After 72 h, cells were washed twice with Dulbecco’s phosphate-buffered saline (DPBS) (without calcium chloride and magnesium chloride; Sigma Aldrich, D8537) and fixed at 4°C with 4% paraformaldehyde solution in phosphate-buffered saline (Santa Cruz Biotechnology, sc-281692) for 15 min. Cells were then washed and incubated for 30 min at RT with DPBS blocking solution supplemented with 2% goat serum (Sigma Aldrich, G9023) and 0.2% Tween 20 (AppliChem, A1389). The primary antibodies, mouse anti-myosin heavy chain (MF20, 1/1000) (MF 20 was deposited to the DSHB by D.A. Fischman, DSHB Hybridoma Product MF 20) and rabbit anti-MEF2 (1/300) (Santa Cruz Biotechnology, sc-313), were prepared in blocking solution and added to the cultures for overnight incubation at 4°C. After incubation, cells were washed three times with DPBS and incubated with blocking solution for 5 min. The secondary antibodies, (1/1,000) goat anti-mouse Alexa 488 (Life Technologies, A11029) and goat anti-rabbit Alexa 546 (Life Technologies, A11035), were prepared in blocking solution and added to the cultures for 1 h at RT. After three washes with DPBS, coverslips were mounted with DAPI Fluoromount-G Mounting Medium (SouthernBiotech, 0100-20) and examined under a fluorescence microscope (20× objective). For the quantification of differentiation percentage and fusion index, seven pictures per condition were randomly acquired. The number of MEF2-positive nuclei and DAPI-stained nuclei were quantified to calculate the fusion index.

### Myoblast cell culture in hypoxic conditions

Cell cultures were plated at a density of 2 × 10^5^ cells/mL in a 60-mm Petri dish containing 3.5 mL of GM and incubated for 24 h as described above. Culture dishes were then placed in a hypoxic incubator chamber containing 1% oxygen, at 37°C, for 24 h.

### Hollow fiber capsules and myoblast encapsulation

For macroencapsulation,10-mm-long hollow fiber capsules were prepared as described previously^44^ using a modified polyethersulfone porous membrane with 0.65 μm pore size and 0.75 mm inner diameter (Spectrum labs) containing polyester yarns (44/27-PET-5540-FTT-SS, Textile Development Associates).

Cell cultures were plated at a density of 2 × 10^5^ cells/mL in a 60-mm Petri dish containing 3.5 mL of GM and incubated for 24 h as described above. Cells were detached with trypsin, counted in duplicate using an automated cell counter (Countess II device, Thermo Fisher Scientific) and resuspended in the GM. A total of 25 μL of the cell suspension was loaded into each capsule through a vasculon (loading tube). When the loading was complete, the vasculon was cut and capsules were sealed using UV-Sensitive Medical Device Adhesive (Dymax, 1187-M-SV01). Capsules were placed in 12-well plates with 2 mL of GM for 24 h before implantation.

### Freezing and thawing of capsules loaded with human myoblasts

Before freezing, capsules were loaded using the same procedure, but with cells resuspended in freezing medium (GM supplemented with 10% glycerol [Sigma Aldrich, 49,767]) and immediately placed in silicone tubes prefilled with freezing medium, after which they were transferred to cryotubes. The cryotubes were quickly transferred to a CoolCell freezing container (Corning) and frozen at −80°C overnight. For long-term storage, cryotubes were conserved in liquid nitrogen.

The thawing process involved rapidly transferring the frozen capsules in silicone tubes from liquid nitrogen to pre-warmed GM in a 10-cm Petri dish. After gentle agitation, capsules were removed from the silicone tubes and placed in 12-well plates containing 1 mL GM.

### Implantation of hollow fiber capsules in mice

All animals used in this study were housed in a pathogen-free environment with standard temperature and humidity conditions in a 12-h light/dark cycle environment with free access to food and water. All experiments were performed according to local animal care regulation. The study protocol was reviewed and approved by the Committee for Animal Research Ethics at the University of Geneva and by the Cantonal Committee for Animal Experimentation, as per Swiss Federal law (study protocols no. GE/142/17 and GE/97/120).

In brief, adult Rag2/Il2rg double-knockout mice (Taconic Biosciences) received a subcutaneous injection of 0.1 mg/kg buprenorphine for analgesia 20 min before anesthesia. Afterward, mice were anesthetized with isoflurane and capsules were implanted in the subcutaneous tissue of the flank using a trocar. The wound was sutured with surgical staples and animals recovered in their home cage. During this period, 2 mg/mL acetaminophen was added to the mice drinking water for 3 days for analgesia. Mice were sacrificed with a lethal injection of sodium pentobarbital. Capsules were dissected and retrieved from the mice and were either fixed for further histological analysis or placed in culture medium for further quantification of recombinant protein expression. Histological analysis as well as hematoxylin and eosin staining were performed as described previously.^62^

### Implantation of hollow fiber capsules on an *ex vivo* human skin model

The HypoSkin model from Genoskin was used as an *ex vivo* human skin model. This model is based on NativeSkin technology and comprises epidermis, dermis, and subcutaneous fat of human origin embedded in a proprietary solid matrix, with the epidermal surface in direct contact with ambient air. The HypoSkin model is mounted on cell culture inserts, loaded in multi-well companion plates with the lid containing Genoskin medium. This medium is free of serum, growth factors, phenol red, or hydrocortisone. The plates were then kept at 37°C in a standard incubator (5% CO_2_). Capsules were implanted in the subcutaneous tissue using a trocar and punch biopsies were collected at different time points.

### Tissue preparation and cytokine analysis

Punch biopsies were mechanically dissociated in gentleMACS M Tubes (Miltenyi Biotech, 130-093-236) using the Gentle MACS protein program. Tissue samples were processed in 500 μL of T-PER Tissue Protein Extraction Reagent (Thermo Fisher Scientific, 78510) and cOmplete, Mini, EDTA-free Protease Inhibitor Cocktail Tablets (Sigma Aldrich, 04693159001). After dissociation, samples were centrifuged and the supernatant was collected and analyzed for protein concentration. Protein concentration was determined using the Microassay BCA Protein Assay Kit (Thermo Fisher Scientific, 23235) using BSA as the standard. Tissue lysates were analyzed with the V-PLEX Plus Human GM-CSF Kit (Mesoscale Discovery, K151RIG). Protocols were applied according to the manufacturer’s instructions and results were reported in pg/μg total protein.

### ELISA quantification of recombinant protein release

For recombinant protein release analysis, cells were cultured in a T75 cm^2^ culture flask. Once 75% confluency was reached, cells were washed with HBSS and incubated in 5 mL of fresh GM for 2 h at 37°C in a standard incubator. The medium was then collected for quantification and cells were counted using an automated cell counter. Three ELISA kits were used for supernatant analysis: a GM-CSF Human ELISA Kit with a lower limit of quantification at 7.8 pg/mL (Thermo Fisher Scientific, KHC2011), a GM-CSF Mouse ELISA Kit (Thermo Fisher Scientific, BMS612), and an IgG (Total) Human ELISA Kit (Thermo Fisher Scientific, BMS2091). All kits were used according to the manufacturers’ instructions. Results were reported in pg/cell/day.

### GM-CSF potency assay

The potency assay for secreted GM-CSF was evaluated by the LanthaScreen STAT5 TF-1 cellular assay (Thermo Fisher Scientific). This analysis was outsourced to the Thermo Fisher Scientific platform and the protocol was applied according to the manufacturer’s instructions. The GM-CSF sample concentration was quantified by ELISA. The potency capacity was directly correlated with the EC_50_ and was determined with a 10-point titration curve. Recombinant GM-CSF was used as a positive control. To compare potency in the screening assay, the Z-factor was calculated as described previously.^74^

### Chromatography with FL detection

Measurements were performed on an Acquity UPLC I-Class system (Waters, Milford, MA, USA) equipped with a binary solvent delivery pump, an autosampler, and an FL detector. For all measurements, a sample volume of 10 μL was injected and FL detection (excitation at 280 nm, emission at 340 nm) was applied. Data acquisition and instrument control were performed by Empower Pro 3 software (Waters). The stationary phase employed for the RPLC analysis was a Waters Bioresolve RP mAb polyphenyl (2.1 × 150 mm, 2.7 μm). Mobile phase flow rate and column temperature were set at 500 μL/min and 80°C, respectively, while the mobile phase consisted of water + 0.1% trifluoroacetic acid (A) and acetonitrile + 0.1% trifluoroacetic acid (B). The applied gradient was the following: 0 min, 30% B; 1 min, 30% B; 11 min, 40% B; 14 min, 80% B; 15 min, 80% B; 15.1 min, 30% B; 25 min, 30% B. DL-dithiothreitol (DTT), and trifluoroacetic acid were purchased from Sigma-Aldrich (Buchs, Switzerland). Water and acetonitrile of UPLC-MS grade were purchased from Fisher Scientific (Reinach, Switzerland). Ipilimumab was obtained as European Union pharmaceutical-grade drug products from its respective manufacturer (Bristol Myers Squibb). A stock solution was prepared by diluting 20 μg/mL of ipilimumab with water. Intact ipilimumab sample (100 μL) was obtained by diluting 50 μL of stock solution at 10 μg/mL with water and injected without further preparation. Reduced ipilimumab sample (100 μL) was prepared by adding 50 μL of 0.04 M DTT solution to 50 μL of the mAb solution (20 μg/mL). The resulting solution at 10 μg/mL of ipilimumab was finally incubated at 37°C for 30 min. The 100-μL sample was then then transferred to HPLC vial for the analysis.

### Competition assay for CD20 binding

The supernatant collected from myoblast cultures secreting anti-CD20 antibody was analyzed in a binding capacity assay. Rituximab (anti-CD20, Hoffmann-La Roche) was used as a binding positive control. Human PBMCs isolated from whole blood using BD Vacutainer CPT Sodium Citrate tubes (BD Biosciences, 362782) were pre-incubated with different concentrations of either supernatant or rituximab (8–0.25 μg) for 30 min at 4°C. Cells were then washed twice with DPBS and stained for 30 min at 4°C with anti-CD20 FITC (BD Biosciences, 555622) and anti-CD19 PE-CF594 (BD Biosciences, 562321). DRAQ7 staining was used (BD Biosciences, 564904) for the selection of viable cells. Stained cells were analyzed using an LSR Fortessa flow cytometer (BD Biosciences) and data were analyzed using BD FACS Diva (BD Biosciences).

### Checkpoint inhibitor (CTLA-4) blockade assay

The CTLA-4 blockade assay was outsourced to GenScript Biotechnology (Nanjing). This assay was performed with the CTLA-4 Blockade Bioassay kit (Promega, JA3001) according to the manufacturer’s instructions. Culture supernatant was used as the source of myoblast-secreted anti-CTLA4. No concentration step was used to avoid altering the activity of the antibody and be as close as possible to the conditions when it is delivered by encapsulated cells *in vivo*. As a consequence, the highest possible concentration for myoblast-derived material was not as high as the reference material (Yervoy biosimilar) and the concentrations tested were slightly different. Antibody concentration in the sample was quantified as described above for recombinant protein release. Blockade capacity was directly correlated with the EC_50_ and determined with an eight-point titration curve.

### Detection of SARS-CoV-2 modified spike protein

The detection of the SARS-CoV-2 spike protein was performed using three different supernatant dilutions (1, 1/5, and 1/25) collected from encapsulated and non-encapsulated myoblasts secreting the spike protein, which were applied on a PVDF membrane. GM was used as a negative control, whereas the purified spike protein (EPFL, Lausanne) was used as a positive control. The PVDF membrane was incubated in DPBS blocking solution containing 3% non-fat dry milk (Applichem, A0830.0500) for 10 min at RT. Afterward, the membrane was washed three times with DPBS with 0.1% Tween 20 and incubated with the primary antibody (1 μg/mL AQ806, Geneva University’s Antibodies platform) in blocking solution at RT for 2 h with agitation. After incubation, the membrane was washed three times with DPBS with 0.1% Tween 20. Incubation with the secondary antibody diluted 1/1,000 in blocking solution (rabbit anti-mouse horseradish peroxidase; Geneva University’s Antibodies platform) was performed for 1 h at RT. Finally, the membrane was developed using the SuperSignal West Pico PLUS Chemiluminescent Substrate (Thermo Fisher Scientific, 34577). Images were acquired using a Syngene PXi reader.

### Statistical analysis

Results are expressed as mean ± standard deviation (SD). Statistical significance was assessed by two-way analysis of variance (ANOVA) followed by Tukey’s test for analysis of multiple groups, or by Student’s t test for comparison between two groups. Statistical significance between groups is presented as follows: ∗p < 0.05, ∗∗p < 0.01, ∗∗∗p < 0.001, and ∗∗∗∗p < 0.0001. Data analyses were performed using the software package GraphPad Prism 9 (GraphPad Software).

### Data availability

The data required to reproduce these findings are fully available upon request.

## Supplemental information

Document S1. Figures S1–S5 and Tables S1 and S2

Document S2. Article plus supplemental information
