# Immortalized human myoblast cell lines for the delivery of therapeutic proteins using encapsulated cell technology

**DOI:** 10.1016/j.omtm.2023.101130

**Published:** 2023-10-12

**Authors:** Aurelien Lathuiliere, Remi Vernet, Emily Charrier, Muriel Urwyler, Olivier Von Rohr, Marie-Claude Belkouch, Valentin Saingier, Thomas Bouvarel, Davy Guillarme, Adrien Engel, Patrick Salmon, Thomas Laumonier, Julien Grogg, Nicolas Mach

## Main text

(Molecular Therapy: Methods & Clinical Development *26*, 441–458; September 2022)

In the originally published version of this article, the plasma concentration of anti-CD20 was provided in pg/mL in the text and in the graph in Figure 6D. After verifying the source data, the unit has been replaced by ng/mL in both the text and the figure. This has been corrected online.

The authors apologize for this error and any confusion it may have caused.

**Figure:**
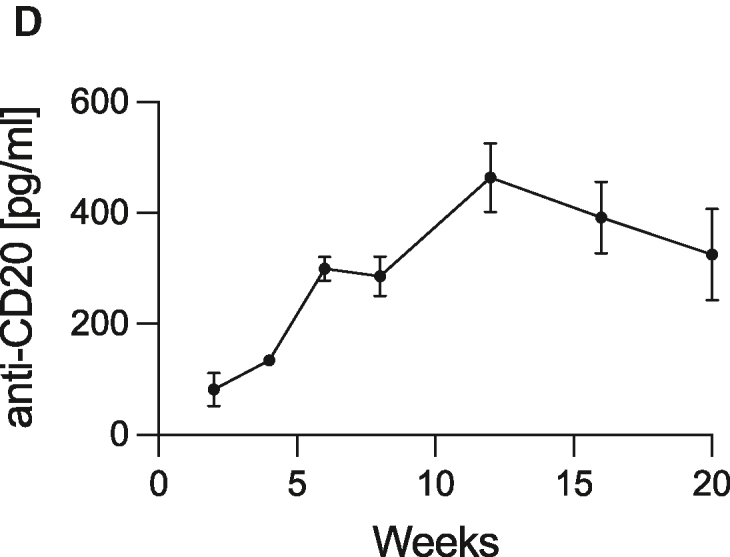
Figure 6D. Novel immortalized myoblasts can be used for the continuous delivery of complex therapeutic macromolecules with preserved functionality (original)

**Figure:**
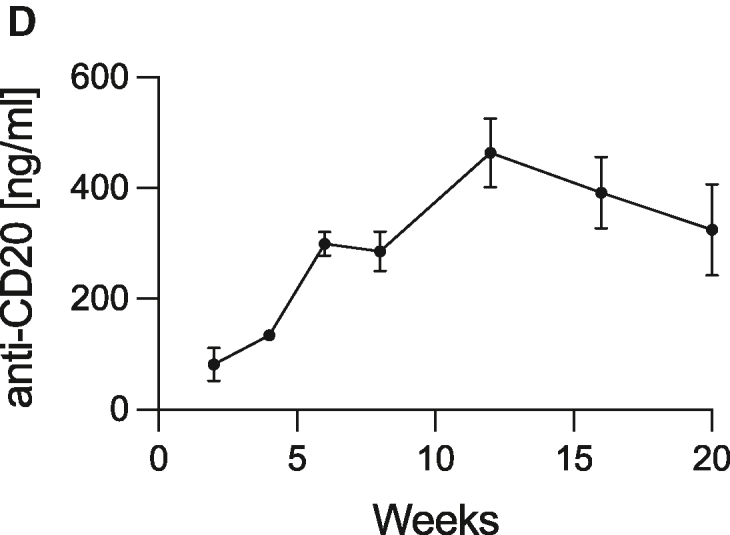
Figure 6D. Novel immortalized myoblasts can be used for the continuous delivery of complex therapeutic macromolecules with preserved functionality (corrected)

