# Supplementary material for: Immortalized human myoblast cell lines for the delivery of therapeutic proteins using encapsulated cell technology
Source: Mol Ther Methods Clin Dev. 2022 Aug 1;26:441–58. doi: 10.1016/j.omtm.2022.07.017 (PMC9418741; doi:10.1016/j.omtm.2022.07.017)
Supplement: Document S1. Figures S1–S5 and Tables S1 and S2 [file mmc1.pdf]

OMTM, Volume *26*

Supplemental information

Immortalized human myoblast cell lines for the delivery of therapeutic proteins using encapsulated cell technology

Aurelien Lathuiliere, Remi Vernet, Emily Charrier, Muriel Urwyler, Olivier Von Rohr, Marie-Claude Belkouch, Valentin Saingier, Thomas Bouvarel, Davy Guillarme, Adrien Engel, Patrick Salmon, Thomas Laumonier, Julien Grogg, and Nicolas Mach

SUPPLEMENTARY MATERIAL

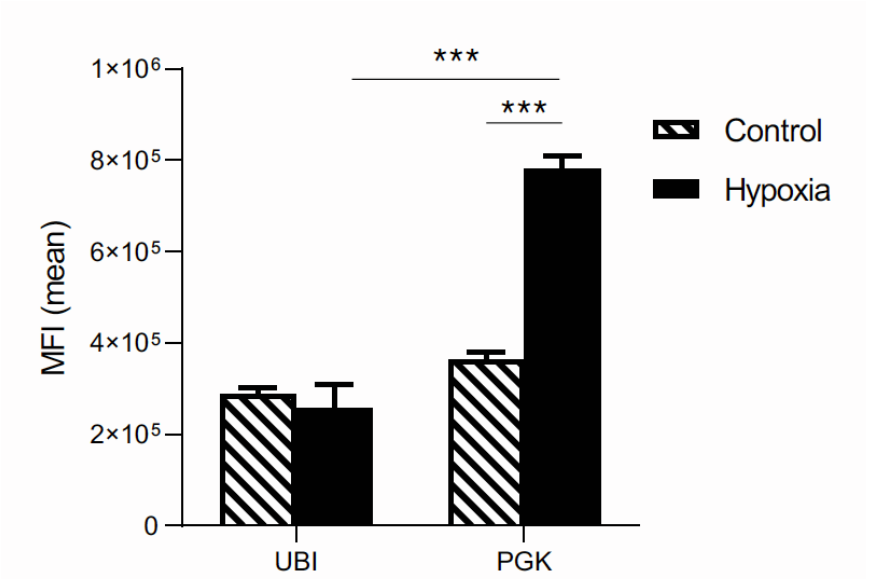

Supplementary Figure S1: The expression of transgenes controlled by the human PGK promoter is upregulated under hypoxic conditions in human myoblast cells. Expression of transgenes controlled by human PGK in hypoxia versus control condition, P= 0.0007. Expression of transgenes controlled by either UBI or PGK promoter in hypoxia conditions, P= 0.0003. Data and errors bars represent mean ± S.D. from two independent experiments. This analysis was performed with a two-way ANOVA Tukey's multiple comparison test.

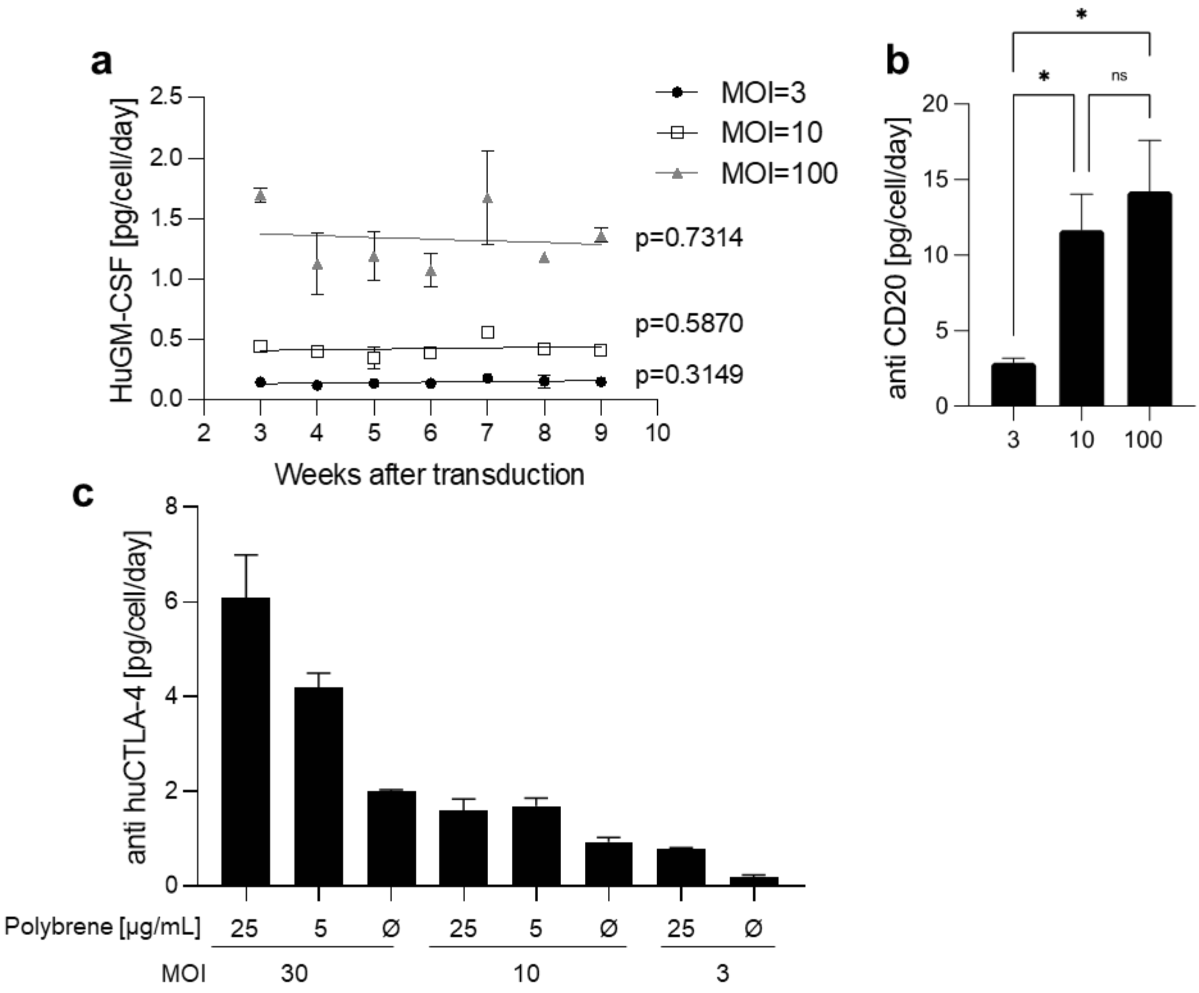

Supplementary Figure S2: The conditions of transduction affect the quantity of molecules secreted by immortalised myoblasts.

Clone #2 parental population was transduced with different multiplicity of infection (MOI) of lentivirus for the expression of huGM-CSF. Each point represents the mean ± SD of two biological replicates. The lines represent a linear regression with p-value for slope not different from zero (a) of human anti-CD20, with each bar representing the mean of two biological replicates. One-way ANOVA (b) and human anti-CTLA-4 (c). The secretion of molecules of interest by the parental population was quantified by ELISA, with each bar representing the mean of two biological replicates.

a

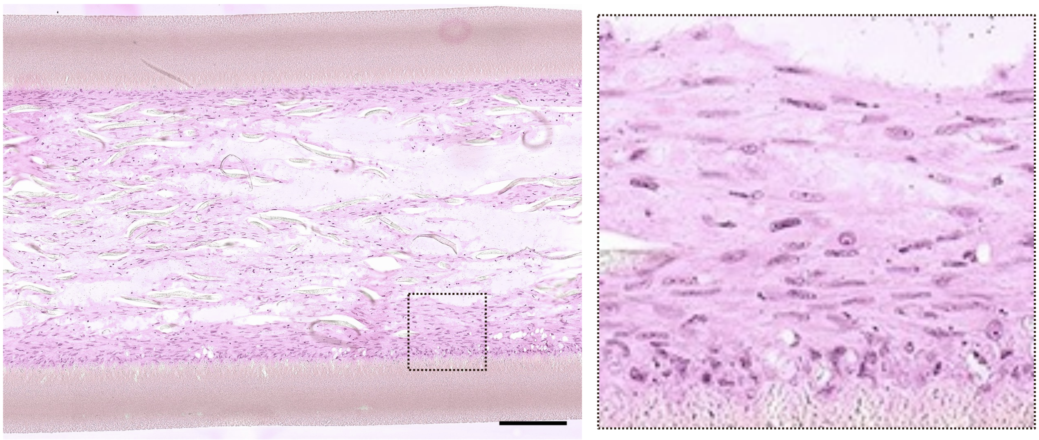

b

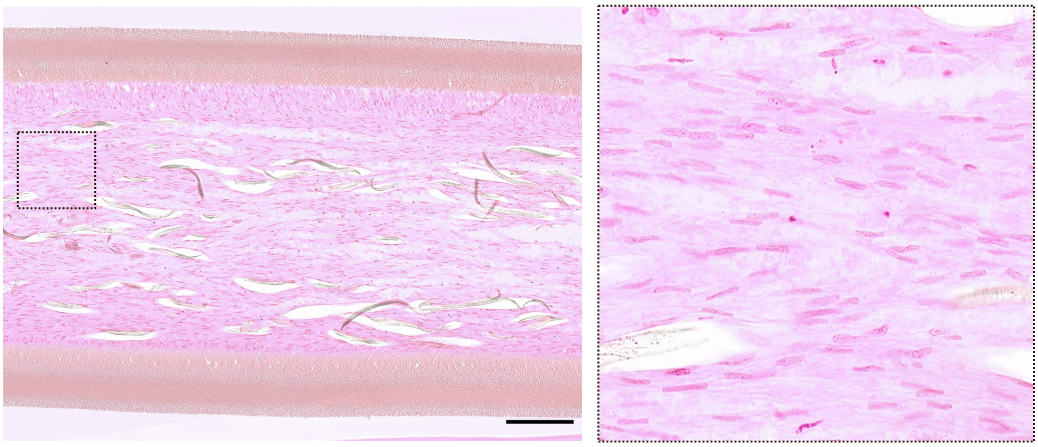

Supplementary Figure S3: *In vitro* evaluation of encapsulated parental immortalized myoblast clone (clone #2). Histological sections of capsules after 1 (a) and 3 (b) weeks *in vitro* demonstrated survival of encapsulated cells at high density with the formation of a dense tissue-like structure. Hematoxylin and eosin staining. Panel on the right is a magnification of the dashed line frame. Scale bar 250um.

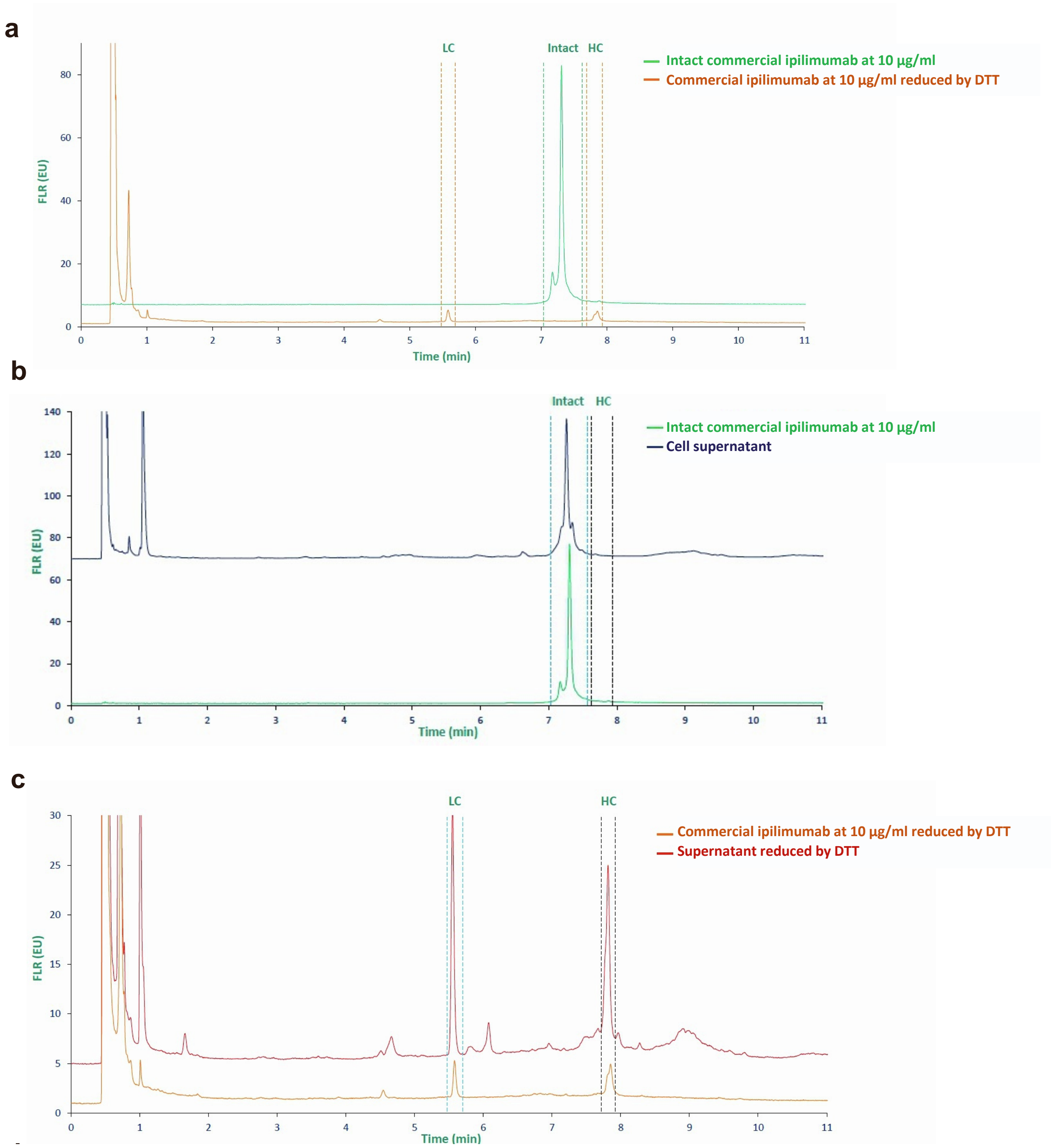

Supplementary Figure S4: Characterization of the secreted anti-CTLA4 heavy and light chains from transduced myoblasts. RPLC-FL chromatograms of intact or reduced samples were obtained to confirm the localization of the free HC and LC and the full IgG on the chromatogram. a. Commercial ipilimumab was run intact or fully reduced. b. The supernatant from myoblasts was compared to intact ipilimumab to confirm that the observed peak corresponds to a full IgG. c. Both samples were fully reduced by DTT. d. Recapitulative table indicating the relative abundance of each protein in the various tested samples.

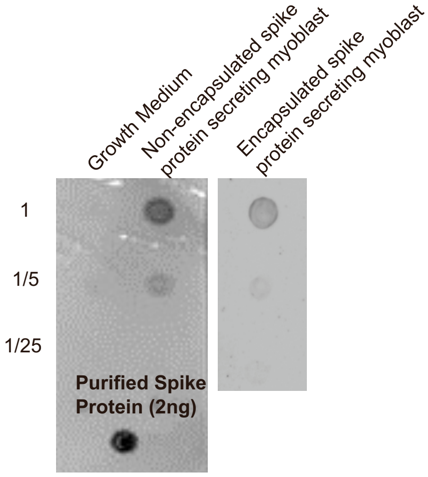

Supplementary Figure S5 : Immortalized clone #2 was transduced with lentivirus to express the SARS-CoV2 spike protein. The secretion of the spike protein by non-encapsulated and encapsulated myoblasts was assessed by dot blot in supernatant.

Supplementary Table 1: Recapitulative table indicating the relative abundance in RPLC-FL chromatograms of each protein in the various tested samples.

	Type	Retention time (min)	Area (µV.sec)	% Area
Intact ipilimumab (10 µg.ml^{-1})	Intact	7.31	3230246	100
Ipilimumab DTT Reduced (10 µg.ml^{-1})	LC	5.58	110381	35.59
	HC	7.85	32178	64.41
Cell supernatant	Intact	7.26	3701436	99.64
	HC	7.69	13480	0.36
Cell supernatant DTT reduced	LC	5.55	738060	39.86
	HC	7.82	1112968	60.13

Supplementary Table 2: Characterization of GM-CSF secreting myoblast line (master cell bank).
MCB: Master Cell Bank; Ph. Eur. : European Pharmacopeia; USP: United States Pharmacopeia; CPE: Cytopathic Effect; Hd: Hemadsorption; Hg: Hemagglutination; F-PERT: Fluorescent Product-Enhanced Reverse Transcriptase.
[a]An out of specification (OOS) investigation was conducted, and concluded that the cause of (rapid) death seen with the adult mice is due to a matrix-associated phenomenon, and not virus contamination of the MVX-2 MCB. This 'matrix interference' disqualifies the utility of the *in vivo* test method using adult mice as fit for purpose for evaluating the MVX-2 MCB for viral safety. Therefore, the OOS investigation was concluded.

Attribute	Test	Acceptance criteria	Results
			**MCB (batch 0A0030)**
**Viability**	Viability, recovery and counting	Viability – Day 0 >80%	Conform
**Cell count**	Viability, recovery and counting	Counting Day 0>70% of $2.2x10^6$ viable cells/ml with a standard deviation of the counting method of 15%	Conform
**Identity**	DNA fingerprinting	-Confirmation of human identity of the cell line<br>-No indications that the sample was contaminated with another human or closely related species cell lines	Conform
**Sterility**	Filtration method (Ph. Eur.2.6.1)	No growth of microorganisms detected	Conform
**Mycoplasma**	Detection of cultivable and non-cultivable mycoplasma (Ph. Eur 2.6.7, USP<63>)	No mycoplasma detected	Conform
**Viral contaminants**	In vitro detection of viral contaminants using 3 sensitive cell lines (Vero, MRC5, HeLa), for CPE and 2 hemadsorption / hemagglutination tests	No extraneous agent detected (i.e. no CPE, no Hd, and no Hg)	Conform
**Adventitious agents**	In vivo detection of viral contaminants using embryonated hen eggs	No evidence for the presence adventitious viruses	Conform
	In vivo detection of viral contaminants using suckling mice	No evidence for the presence adventitious viruses	Non valid[a]
	In vivo detection of viral contaminants using adult mice	No evidence for the presence adventitious viruses	Conform
	Transmission electron microscopy for detection of adventitious agent in cells	-The preparation shows viable cells without any sign of adventitious agent<br>-No virus or virus-like particle could be identified in the cell cytoplasm, nucleus or endoplasmic reticulum at low or high magnification	Conform
**Vector infectivity**	F-PERT detection of reverse transcriptase activity	No retrovirus detected (no Cp with no amplification curve in all tested dilutions)	Conform
**Bovine viruses**	In vitro assay for the detection of bovine viruses in Vero and BT sensitive cell lines	No bovine viral contaminants detected (i.e. no CPE, no Hd, and no specific immunofluorescence	Conform
